# An MHC-Related Gene’s Signature Predicts Prognosis and Immune Microenvironment Infiltration in Glioblastoma

**DOI:** 10.3390/ijms26104609

**Published:** 2025-05-12

**Authors:** Caiyuan Yu, Mingjuan Xun, Fei Yu, Hengyu Li, Ying Liu, Wei Zhang, Jun Yan

**Affiliations:** 1 School of Pharmacy, Faculty of Medicine & State Key Laboratory of Quality Research in Chinese Medicines, Macau University of Science and Technology, Macau SAR 999078, China; yucy@ouchn.edu.cn (C.Y.); 3220004386@student.must.edu.mo (F.Y.); 3220005009@student.must.edu.mo (H.L.); yingliu@xhsysu.cn (Y.L.); 2 Laboratory of Brain Disorders, Beijing Institute of Brain Disorders, Ministry of Science and Technology, Collaborative Innovation Center for Brain Disorders, Capital Medical University, Beijing 100069, China; mingjuan_immunity@163.com; 3 College of Agroforestry and Medicine, The Open University of China, Beijing 100039, China

**Keywords:** glioblastoma, MHC, riskscore, immune microenvironment

## Abstract

Glioma is the most common primary malignant intracranial tumor with limited treatment options and a dismal prognosis. This study aimed to develop a robust gene expression-based prognostic signature for GBM using the Cancer Genome Atlas (TCGA) and Chinese Glioma Genome Atlas (CGGA) datasets. Using WGCNA and LASSO algorithms, we identified four MHC-related genes (TNFSF14, MXRA5, FCGR2B, and TNFRSF9) as prognostic biomarkers for glioma. A risk model based on these genes effectively stratified patients into high- and low-risk groups with distinct survival outcomes across TCGA and CGGA cohorts. This signature correlated with immune pathways and glioma progression mechanisms, showing strong associations with immune function and tumor microenvironment infiltration patterns. The risk score reflected tumor microenvironment remodeling, suggesting its prognostic relevance. We further propose I-BET-762 and Enzastaurin as potential therapeutic candidates for glioma. In conclusion, the four-gene signature we identified and the corresponding risk score model constructed from it provide valuable tools for the prognosis prediction of glioblastoma multiforme (GBM) and may guide personalized treatment strategies. The least absolute shrinkage and selection operator (LASSO) risk score has demonstrated significant prognostic evaluation utility in clinical GBM patients, bringing potential implications for patient stratification and the optimization of treatment regimens.

## 1. Introduction

Glioblastoma multiforme (GBM) is widely recognized as a highly aggressive form of cancer and is the most common primary malignant tumor in the central nervous system (CNS). Epidemiological studies have shown that it imposes a heavy clinical burden, accounting for approximately 14.5% of all central nervous system tumors and nearly half (48.6%) of the cases of malignant tumors in the central nervous system. The prognosis remains far from optimistic. According to contemporary clinical studies, the median survival period of affected patients is typically 15 months after diagnosis [1,2]. GBM is the most aggressive diffuse glioma (Grade IV) originating from the astrocytic lineage. By the time of diagnosis, the tumor has already infiltrated widely, making complete surgical resection almost impossible and greatly increasing the difficulty of treatment. Current treatment regimens for glioblastoma multiforme (GBM) usually adopt a multimodal approach, combining neurosurgical intervention with adjuvant radiotherapy and chemotherapy. However, judging from the discouraging survival data, their clinical efficacy is limited. Epidemiological data show that less than 7% of patients can survive for more than five years after diagnosis [3]. These persistent treatment challenges highlight the urgency of developing reliable prognostic stratification systems and validating molecular markers with diagnostic and therapeutic potential in the field of neuro-oncology.

GBM is characterized by a uniquely complex and immunosuppressive tumor microenvironment that not only drives disease progression but also confers remarkable resistance to current therapeutic interventions [4,5]. The tumor microenvironment (TME) is a complex ecosystem comprising cancer cells, immune cells, endothelial cells, cancer-associated fibroblasts, and various non-cellular components [6]. Within the TME, the process of tumor immune surveillance is crucial for the recognition and elimination of emerging tumor cells, relying heavily on the function of major histocompatibility complex class (MHC) molecules. MHC-I plays a pivotal role in initiating the tumor immune response by presenting specific antigens to CD8+ T cells, thus activating the adaptive immune system [7]. However, overexpressed MHC-I may send inhibitory signals to CD8+ T cells, NK cells, and TAMs, leading to immunosuppressive effects and facilitating tumor survival [8,9,10,11,12,13]. Tumors can also present antigens on MHC-II for recognition through CD4+ T cells, and this pathway could also be subject to regulation for immune evasion [14,15]. Emerging evidence in neuroimmunology indicates that upregulation of major histocompatibility complex (MHC) components, specifically MHC class I and II isoforms, may orchestrate immunoregulatory signaling within glioblastoma microenvironments. Mechanistic studies reveal these molecules functionally modulate cytotoxic T lymphocyte (CTL) activation thresholds and macrophage polarization states—critical determinants of the immune-evasive niche characteristic in GBM pathogenesis. Such immunological perturbations correlate with enhanced tumor immune escape mechanisms and disease progression [15]. To address the unmet clinical need for immunophenotype-driven prognostication, we pioneered the construction of an MHC-centric prognostic signature through integrative computational biology. Our analytical pipeline synergized weighted gene co-expression topology mapping with LASSO-penalized Cox regression, identifying four MHC-interactive biomarkers (TNFSF14, MXRA5, FCGR2B, and TNFRSF9) with prognostic relevance. The risk score model constructed from these genes demonstrates excellent predictive accuracy for overall survival (OS), enabling the precise binary classification of patients and their assignment into distinct survival cohorts with significant differences. This molecular classification system provides a solid theoretical framework and practical guiding basis for the formulation of immunotherapy checkpoint inhibitor treatment regimens and the optimization of immunotherapy strategies in the practice of neuro-oncology.

## 2. Results

### 2.1. Co-Expression Network and Module Identification Reveal MHC-Related Gene Clusters in Glioblastoma

**Figure 1:**
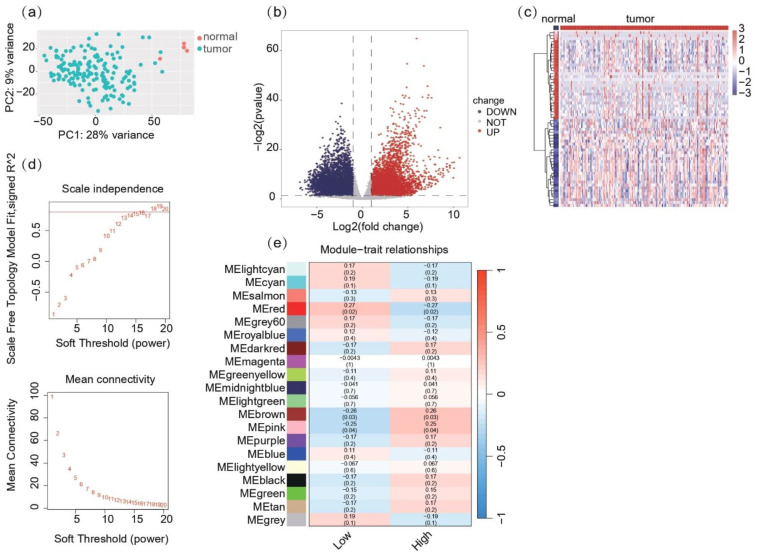
Co-expression network construction and identification of MHC-related modules in glioblastoma: (**a**) principal component analysis (PCA) of GBM and normal samples. PCA plot of variance-stabilized RNA-seq data from 168 GBM and 5 normal tissue samples. Samples are color-coded based on tissue type: GBM tumor (red) and normal (blue). The first two principal components are shown, accounting for the largest variance across the dataset, clearly separating tumor and normal samples; (**b**) volcano plot of differentially expressed genes (DEGs) between GBM and normal tissues. Volcano plot showing log_2_fold change (*x*-axis) versus −log_10_ adjusted *p*-value (*y*-axis) for all genes analyzed. Upregulated genes (log2FC > 1, adjusted *p*-value < 0.05) are colored in red, and downregulated genes (log2FC < −1, adjusted *p*-value < 0.05) are in blue. Non-significant genes are shown in gray. Dashed lines represent significance thresholds for fold change (log2FC = ±1) and *p*-value (adjusted *p*-value = 0.05); (**c**) heatmap of the top 60 differentially expressed genes in GBM vs. normal tissues. Heatmap showing expression levels (log2-transformed counts) of the top 30 upregulated and 30 downregulated genes in GBM compared to normal tissues. Samples are clustered based on gene expression patterns, with GBM tumor samples (*n* = 168) labeled in red and normal samples (*n* = 5) in blue. The color scale represents z-scores, with red indicating high expression and blue indicating low expression. (**d**) Soft-thresholding power analysis. (Upper figure) plot of scale-free topology model fit versus soft threshold power. The selected power value of 5 is highlighted. (Lower figure) mean connectivity as a function of soft-threshold power. (**e**) Module–Trait relationship. Heatmap showing the correlation between module eigengenes and clinical traits, highlighting significant associations for the red modules with MHC traits.

RNA-seq count data for glioblastoma (GBM) patients were acquired from TCGA as the training set and preprocessed. Using DESeq2, a total of 13,227 genes were analyzed for differential expression between tumor (*n* = 168) and normal (*n* = 5) samples (Figure 1a). Specifically, 1523 genes were significantly upregulated in the GBM tumor samples, while 1089 genes were downregulated (|log2FC| > 1, adjusted *p*-value < 0.05). A volcano plot was generated to visualize the distribution of all DEGs, highlighting significantly upregulated and downregulated genes (Figure 1b). Heatmap analysis of the top 60 DEGs, consisting of the top 30 most upregulated and 30 most downregulated genes, revealed distinct expression patterns between GBM tumors and normal tissues (Figure 1c). Hierarchical clustering indicated clear segregation of tumor and normal samples, supporting the robustness of the DEGs identified in the analysis.

Weighted gene co-expression network analysis (WGCNA) identified 19 gene co-expression modules based on expression data from DEGs and MHC ssGSEA. Hierarchical clustering and outlier detection ensured high-quality data for the network construction (Figure 1d,e). WGCNA revealed that the red module exhibited significant correlations with MHC-related traits, suggesting this module is involved in MHC-related immune processes within the tumor microenvironment. These findings indicate potential MHC-related immune-regulatory gene networks in glioblastoma, providing insights into tumor-immune interactions.

### 2.2. Identification of 4 Prognostic Key Genes Using LASSO Regression Analysis

**Figure 2:**
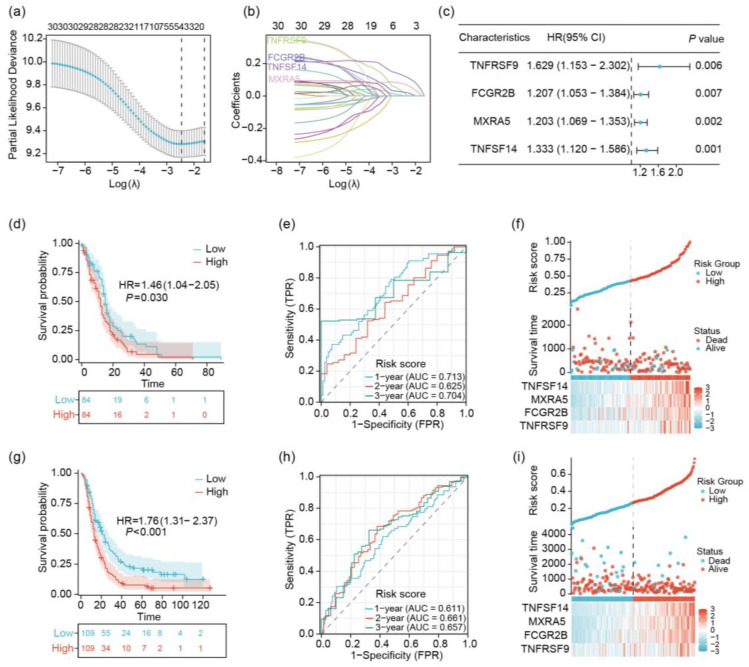
LASSO regression analysis identifies four MHC-related genes as prognostic biomarkers in GBM: (**a**) LASSO coefficient profiles showing the change in coefficient values for all genes considered during LASSO regression as a function of the log-transformed tuning parameter (λ). The optimal λ value was determined by 10-fold cross-validation (see panel **b**). (**b**) Ten-fold cross-validated LASSO paths illustrating the selection and weighting of the four significant MHC-related genes (TNFSF14, MXRA5, FCGR2B, and TNFRSF9) across repeated iterations. Each line represents a single gene’s coefficient path. (**c**) Results from univariate Cox proportional hazards models assessing the prognostic significance of each of the four identified MHC-related genes. Hazard ratios (HRs) with 95% confidence intervals and *p*-values are shown. (**d**–**f**) Analysis of the TCGA training cohort. (**d**) Kaplan–Meier curves for overall survival in high- and low-risk groups. (**e**) Time-dependent ROC curves at 1, 2, and 3 years. (**f**) Risk score distribution, survival status, and expression heatmap of model genes. (**g**–**i**) Validation in the CGGA testing cohort. (**g**) Kaplan–Meier curves for overall survival in high- and low-risk groups. (**h**) Time-dependent ROC curves at 1, 2, and 3 years. (**i**) Risk score distribution, survival status, and expression heatmap of model genes. In (**a**,**d**), patients were stratified into high- and low-risk groups based on the median risk score. *p*-values were calculated using the log-rank test. In (**b**,**e**), the *x*-axis represents the false-positive rate, and the *y*-axis represents the true-positive rate. AUC values are shown for each time point. In (**c**,**f**), the top panel shows the distribution of risk scores, the middle panel displays patient survival status, and the bottom panel presents a heatmap of model gene expression. Red indicates high expression; blue indicates low expression. Patients are ordered by increasing risk score from left to right.

To further explore the role of MHC in gliomas, we used LASSO regression analysis and identified four genes (TNFSF14, MXRA5, FCGR2B, and TNFRSF9) related to the major histocompatibility complex (MHC). These genes were consistently associated with patient survival across 10-fold cross-validation (Figure 2a). The tuning parameter was determined using a repeated 10-fold cross-validation approach (Figure 2b). Moreover, univariate Cox analysis indicated that all four genes were significant prognostic factors (hazard ratios (HRs) > 1 and *p*-values < 0.05) (Figure 2c). Therefore, we utilized the LASSO risk scores derived from these genes to predict the survival of glioblastoma patients. Our analysis of the TCGA training cohort revealed significant prognostic implications of the risk score model. Kaplan–Meier analysis demonstrated a marked difference in overall survival (OS) between high- and low-risk groups (*p* = 0.030, log-rank test). Patients classified as low-risk exhibited substantially improved survival outcomes, with a median OS of 14 months compared to 10 months in the high-risk group (HR = 1.46, 95% CI: 1.04–2.05) (Figure 2c). The predictive accuracy of the risk score model was further validated through time-dependent receiver operating characteristic (ROC) curve analysis. We observed consistently high area under the curve (AUC) values across various time points, with AUCs of 0.713, 0.625, and 0.704 at 1, 2, and 3 years, respectively. The analytical results demonstrate significant prognostic stratification capacity across temporal survival endpoints (Figure 2d). A multidimensional assessment of risk score patterns revealed two principal findings: first, a direct correlation between elevated risk scores and clinical lethality; second, a progressive risk continuum evidenced by incremental score increases rather than distinct categorical divisions (Figure 2e). To establish methodological rigor, we implemented an external validation framework using the Chinese Glioma Genome Atlas (CGGA) cohort. This independent replication confirmed the model’s prognostic fidelity, with Kaplan–Meier survival curves showing marked disparity between risk stratifications (log-rank *p* < 0.001; hazard ratio [HR] 1.76, 95% confidence interval [CI] 1.31–2.37). Survival metrics between cohorts displayed remarkable concordance, with median overall survival durations of 20 versus 13 months distinguishing low- and high-risk populations in both datasets (Figure 2f). Temporally predictive performance assessment through ROC analysis yielded comparable discriminative accuracy in the validation cohort (AUC 0.611/0.661/0.657 at 1/2/3 years), mirroring TCGA-derived metrics (Figure 2g). Cross-cohort consistency extended to risk architecture characteristics, with CGGA analyses replicating the TCGA-observed integration of risk gradients and survival probabilities (Figure 2h). This multi-institutional validation substantiates the model’s biological generalizability, particularly evidenced by preserved prognostic precision across ethnogeographically distinct populations in TCGA and CGGA datasets. The developed risk quantification system thus demonstrates three essential qualities: temporal resolution for survival prediction, mathematical stability across heterogeneous cohorts, and clinical interpretability through continuous risk parameterization.

### 2.3. Analysis of TNFSF14, MXRA5, FCGR2B, and TNFRSF9 in GBM

**Figure 3:**
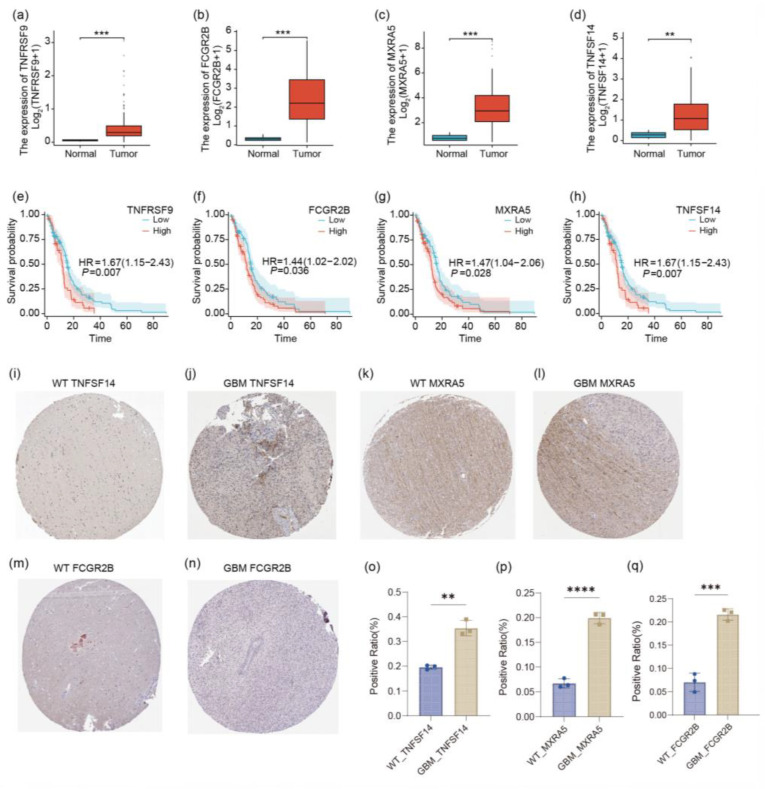
Association of TNFSF14 and MXRA5 expression with GBM prognosis: (**a**–**d**) Differential expression analysis of TNFSF14, MXRA5, FCGR2B, and TNFRSF9 in GBM tumor samples versus normal brain tissue. Box plots depict log_2_(TPM+1) values, with statistical significance determined by unpaired *t*-tests. *p*-values are indicated for each comparison. (**e**–**h**) Kaplan–Meier survival analyses illustrating the prognostic significance of TNFSF14, MXRA5, FCGR2B, and TNFRSF9 expression levels in GBM patients. Hazard ratios (HRs) and corresponding 95% confidence intervals (CIs) are provided for each gene. (**i**–**q**) Representative immunohistochemical staining of TNFSF14 and MXRA5 in GBM and normal brain tissue specimens. Representative immunohistochemical staining of TNFSF14, MXRA5, and FCGR2B in glioblastoma multiforme (GBM) and normal brain tissue specimens, as well as the statistical results of their related immunohistochemical positive rates. **** *p* < 0.0001; *** *p* < 0.001; ** *p* < 0.01.

Our analysis revealed significantly elevated expression levels in the expression profiles of TNFSF14, MXRA5, FCGR2B, and TNFRSF9 in GBM tissues compared to normal brain samples (Figure 3a–d). Kaplan–Meier survival analyses were conducted to evaluate the prognostic significance of these genes in GBM patients (Figure 3e–h). The high expression of all four of these genes was significantly associated with poor overall survival. To further validate our transcriptomic findings, we examined immunohistochemical (IHC) staining data for TNFSF14 and MXRA5 in GBM and normal brain tissue specimens available from the Human Protein Atlas (https://www.proteinatlas.org (accessed on 5 March 2025)) (Figure 3i–l). Consistent with our gene expression analysis, the publicly available IHC data showed that TNFSF14 exhibited strong positive staining in GBM tissues, with notably increased intensity compared to normal brain samples. MXRA5 also demonstrated enhanced staining in GBM specimens. These protein-level data from an independent source corroborate our transcriptomic findings, providing additional support for the differential expression of these genes in GBM. In summary, our findings, supported by both transcriptomic analysis and publicly available protein expression data, highlight the potential roles of TNFSF14 and TNFRSF9 as prognostic biomarkers in GBM. This study underscores the complex relationship between gene expression, protein levels, and clinical outcomes in this aggressive malignancy.

### 2.4. The Risk Signature Is Strongly Associated with Immune Functions in GBM

**Figure 4:**
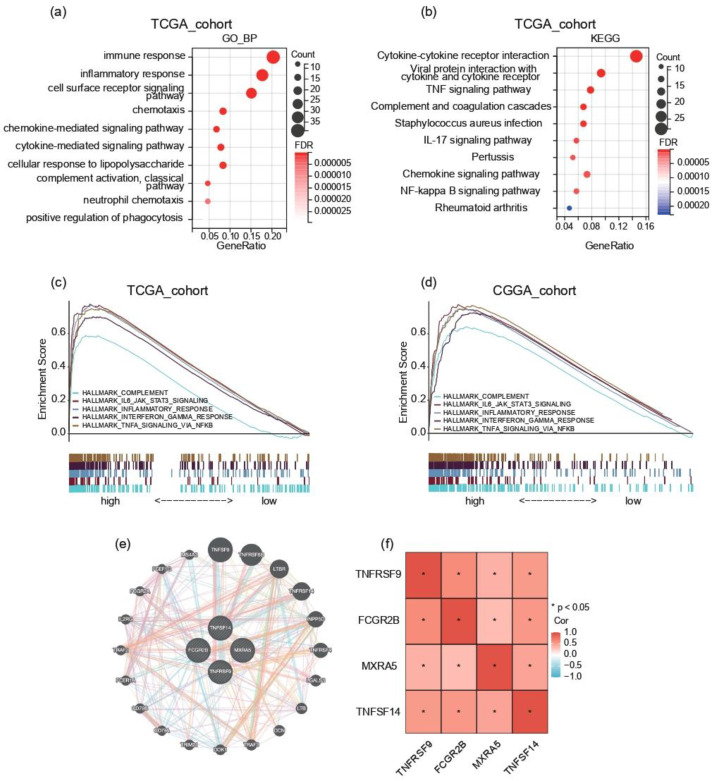
The main functional pathways significantly correlated with risk score: (**a**,**b**) Gene Ontology (GO) enrichment analysis and Kyoto Encyclopedia of Genes and Genomes (KEGG) pathway analysis of upregulated transcripts. This bar chart shows the most significantly enriched GO terms and KEGG pathways associated with the upregulated differentially expressed genes (DEGs) in the TCGA cohort. (**c**,**d**) Enriched gene sets in the HALLMARK collection according to samples with high-risk scores. (**e**) Gene–gene interaction network from GeneMANIA. (**f**) Correlation analysis of key differentially expressed genes. This heatmap visualizes the pairwise Pearson correlation coefficients among selected DEGs of interest (TNFSF14, MXRA5, FCGR2B, and TNFRSF9). The color scale ranges from blue (strong negative correlation) to red (strong positive correlation), with the intensity reflecting the magnitude of the correlation. Statistically significant correlations (*p* < 0.05) are denoted with an asterisk.

Comprehensive transcriptomic characterization through TCGA data delineated distinct molecular signatures differentiating risk-stratified populations. Employing stringent differential expression criteria (adjusted *p* < 0.05, |log2FC| > 1), we identified 218 protein-coding transcripts demonstrating significant elevation in high-risk subjects. To elucidate the systems-level pathophysiological associations of these dysregulated genes, we implemented multilayered functional annotation protocols, encompassing both ontological categorization (Gene Ontology) and pathway topology analysis (KEGG). Using DAVID analysis, these upregulated genes were mainly enriched in the biological processes of ‘immune response’, ‘inflammatory response’, ‘chemokine-mediated signaling pathway’, and ‘adaptive immune response’ (Figure 4a and Figure S1a). The high-risk score group was also enriched in ‘HALLMARK_COMPLEMENT’, ‘HALLMARK_IL6_JAK_STAT3_SIGNALING’, ‘HALLMARK_INFLAMMATORY_RESPONSE’, ‘HALLMARK_INTERFERON_GAMMA_RESPONSE’, and ‘HALLMARK_TNFA_SIGNALING_VIA_NFKB’ in both CGGA and TCGA cohorts using GSEA (Figure 4c,d). These results suggest that the risk score could be a good indicator of the immune response in GBM.

All four MHC-related genes were found among the upregulated genes in the differential analysis. To elucidate the functional interplay among these genes, we examined their gene–gene interaction network and correlation analysis (Figure 4e,f). Our analysis revealed a moderate positive correlation between TNFRSF9 and FCGR2B (r = 0.5978, *p* = 0), TNFRSF9 and TNFSF14 (r = 0.5128, *p* = 1.02 × 10^−12^), and FCGR2B and TNFSF14 (r = 0.5282, *p* = 1.57 × 10^−13^), suggesting potential co-regulation or functional synergy.

### 2.5. The High Risk Score Predicts the Enrichment of Macrophages and CD8+ T Cells in GBM

**Figure 5:**
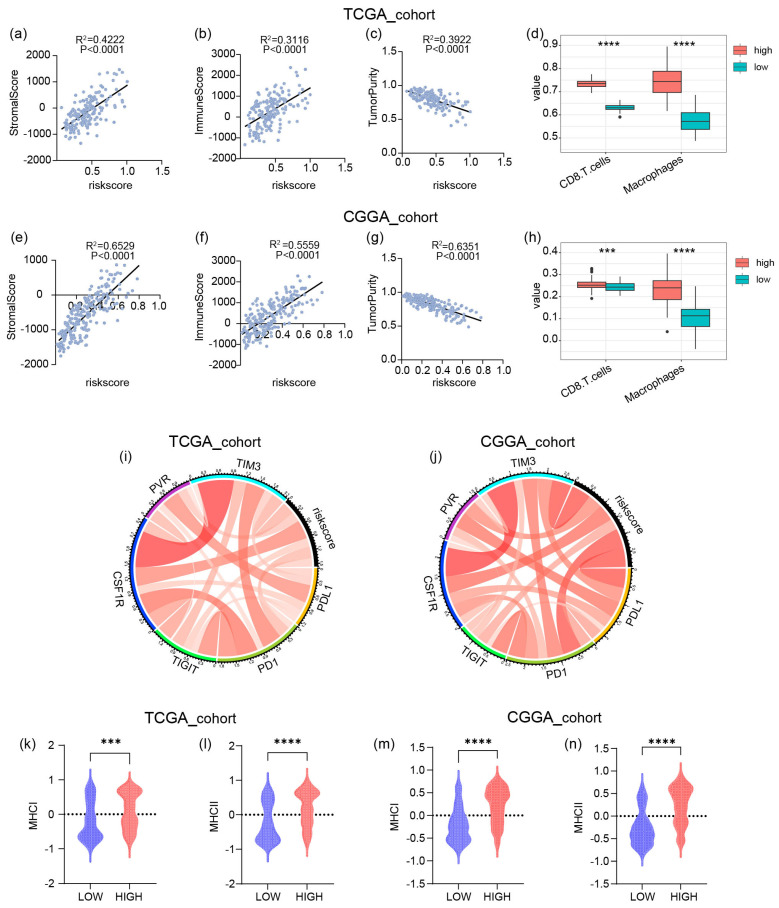
The potential relationship between risk scores and immunity: (**a**–**c**,**e**–**g**) The scatter plots show the relationships between the risk score and the stromal score, immune score, or tumor purity. The risk score is positively correlated with the stromal score and immune score and negatively correlated with the tumor purity. (**d**,**h**) The correlation between immune infiltrating cells and the risk score in TCGA and CGGA cohorts. (**i**,**j**) Pearson correlation analysis between risk score and immune checkpoints in TCGA and CGGA cohorts. (**k**–**n**) The relationships between the risk score and the MHC score. **** *p* < 0.0001; *** *p* < 0.001.

The aforementioned analysis revealed that high risk scores are associated with immunity. To further explore the relationship between risk scores and the tumor microenvironment (TME), we calculated stromal and immune scores for each case in the Chinese Glioma Genome Atlas (CGGA) and the Cancer Genome Atlas (TCGA) datasets (Figure 5a–c,e–g). The results demonstrated positive correlations between both stromal scores and immune scores with risk scores, while tumor purity showed a negative correlation with risk scores. These findings indicate that the proportion of infiltrating immune cells in gliomas increases with higher risk scores. Furthermore, we applied ssGSEA to score the samples and observed that CD8+ T cells and macrophages were also highly expressed in the high-risk score group. These findings led us to hypothesize a potential association between our gene signature and the infiltration of CD8+ T cells and macrophages in gliomas, which may promote immunosuppression. Although immune checkpoint blockade (ICB) combination therapy has demonstrated efficacy in preclinical glioma models, we sought to investigate the relationship between the risk score signature and relevant immune checkpoints. The results revealed that CSF1R showed a strong correlation with the risk score (Figure 5d,h). Our findings suggest that high-risk score gliomas may drive an immunosuppressive microenvironment via the CSF1R-macrophage axis, thereby restricting the anti-tumor functionality of CD8+ T cells and, ultimately, leading to resistance to ICB therapy. Given that the four genes used to construct the risk score are all genes related to the major histocompatibility complex (MHC), in order to deeply explore their potential mechanisms of action and related characteristics in the samples, subsequently, for the samples from the Cancer Genome Atlas (TCGA) and the Chinese Glioma Genome Atlas (CGGA), we employed the single-sample gene set enrichment analysis (ssGSEA) method to conduct a scoring analysis of the MHC-related gene sets. The analysis results showed that a high risk score is usually accompanied by a high MHC score (Figure 5k–n).

### 2.6. Screening GBM Therapeutic Drugs via Critical Genes

**Figure 6:**
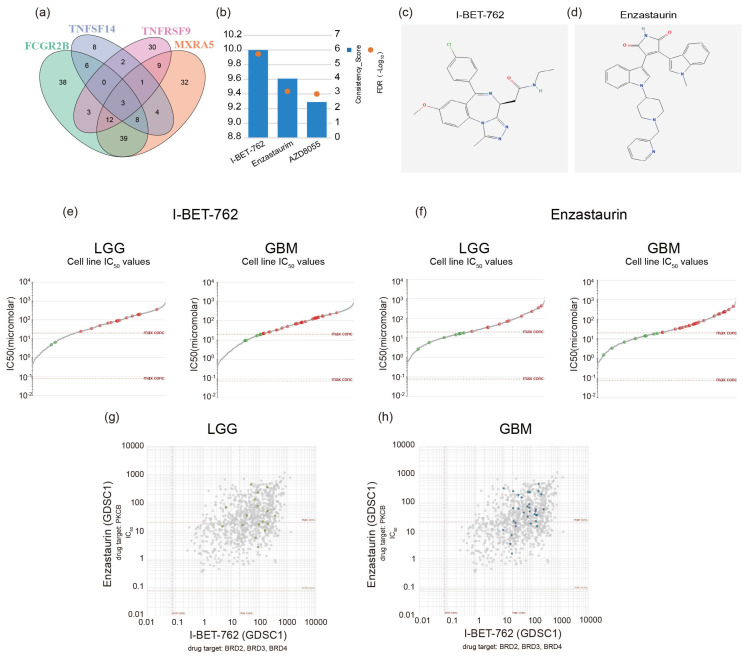
Two potential chemotherapeutic drugs were identified using four key genes: (**a**) The Venn diagram shows that among the four candidate genes significantly correlated with drug efficacy, there is an overlap of three small-molecule compounds as potential drugs. (**b**) Consistency score and FDR value of small-molecule drugs. (**c**,**d**) Structural diagrams of the molecules of two compounds. (**e**,**f**) Half-maximal inhibitory concentration (IC_50_) plots of these two molecules in LGG and GBM. (**g**,**h**) The therapeutic effects of these two small-molecule drugs show a certain correlation in both low-grade glioma (LGG) and high-grade glioma (HGG).

We downloaded a list of small-molecule compounds potentially related to the molecular expression and correlation indices of the key genes (TNFSF14, MXRA5, FCGR2B, and TNFRSF9) from the RNAactDrug database. We selected the top 20 small molecules with the strongest correlation for each gene expression and performed an intersection analysis. As a result, three small-molecule compounds were identified: I-BET-762, Enzastaurin, and AZD8055 (Figure 6a,b). We chose the top two small-molecule compounds with the highest correlations for further study. The molecular structure diagrams were obtained from the ChEMBL database (https://www.ebi.ac.uk/ (accessed on 26 March 2025)) and the HMC LINCS database (https://lincs.hms.harvard.edu/ (accessed on 26 March 2025)) (Figure 6c,d). Drug sensitivity was calculated using the Cancerrxgene database (https://www.cancerrxgene.org/ (accessed on 26 March 2025)). A total of 17 low-grade glioma (LGG) cell lines and 33 glioblastoma (GBM) cell lines were selected for the drug sensitivity test. In LGGs, the maximum, mean, and minimum half-maximal inhibitory concentration (IC_50_) values of I-BET-762 were 360 μM, 70.3 μM, and 4.86 μM, respectively. The maximum, mean, and minimum IC_50_ values of Enzastaurin were 440 μM, 36.3 μM, and 2.67 μM, respectively. In GBMs, the maximum, mean, and minimum IC_50_ values of I-BET-762 were 263 μM, 59.2 μM, and 9.48 μM, respectively. The maximum, mean, and minimum IC_50_ values of Enzastaurin were 458 μM, 47.6 μM, and 1.52 μM, respectively (Figure 6e,f). A positive correlation was observed between the two molecules in both LGGs (R = 0.4021) and GBMs (R = 0.4021) (Figure 6g,h).

## 3. Discussion

Gliomas, particularly glioblastoma (GBM), are among the most aggressive tumors with poor prognoses [16]. Despite extensive efforts to improve survival outcomes, the prognosis remains unfavorable [17]. The standard treatment regimen for gliomas includes maximal surgical resection followed by radiotherapy and temozolomide chemotherapy [18]. However, the limited efficacy of current therapies is largely attributed to immune evasion mechanisms [19]. Consequently, elucidating the role of the immune microenvironment in gliomas and enhancing immunotherapeutic efficacy represent promising avenues for improving patient outcomes.

The major histocompatibility complex (MHC) plays a pivotal role in the tumor immune microenvironment. MHC class I molecules primarily present endogenously processed antigens to CD8+ cytotoxic T lymphocytes, whereas MHC class II molecules mediate antigen presentation to CD4+ helper T cells [20]. Emerging evidence suggests that the role of MHC molecules in cancer is context-dependent, influencing both immune activation and immune suppression. Paradoxically, the upregulation of MHC components has been implicated in tumor immune evasion through multiple pathways. Clinical studies in melanoma and ovarian cancer have demonstrated that MHC class I overexpression is associated with the activation of the programmed death-ligand 1 (PD-L1)/programmed death-1 (PD-1) axis, leading to the functional impairment of cytotoxic T lymphocytes (CTLs) [6,20]. Similarly, the overexpression of MHC class II molecules has been linked to regulatory T cell (Treg) recruitment via CXCL12/CXCR4 signaling, thereby establishing an immunosuppressive microenvironment [15]. These immune editing processes highlight the therapeutic potential of targeting MHC-mediated immune escape pathways. However, the prognostic implications of MHC-associated transcriptional programs in gliomagenesis remain largely unexplored.

To address this knowledge gap, we developed an MHC-centered molecular signature for glioma diagnosis and survival prediction. Using a novel computational framework integrating weighted gene co-expression network analysis (WGCNA) and least absolute shrinkage and selection operator (LASSO) regression, we identified a four-gene prognostic signature comprising tumor necrosis factor superfamily member 14 (TNFSF14), transmembrane protein with X-linking motif 5 (MXRA5), Fc gamma receptor IIB (FCGR2B), and tumor necrosis factor receptor superfamily member 9 (TNFRSF9). This signature demonstrated robust prognostic utility in two independent GBM cohorts: the Cancer Genome Atlas (TCGA) and the Chinese Glioma Genome Atlas (CGGA).

TNFSF14 (CD258/LIGHT) is a transmembrane immunomodulatory protein expressed on activated T cells, natural killer (NK) cells, and dendritic precursor cells, where it influences antigen presentation (MHC-I/II), signal transduction (STAT1/LCK), and myeloid cell activation [21,22,23,24]. MXRA5 exhibits anti-inflammatory and anti-fibrotic properties and has been implicated in tumorigenesis, serving as a potential biomarker for multiple malignancies, including colorectal, lung, pancreatic, and gastric cancers, as well as glioma and acute myeloid leukemia [25,26,27,28]. FCGR2B encodes FcγRIIB, an immunoglobulin G (IgG) receptor expressed on antigen-presenting cells such as B cells, macrophages, and dendritic cells [29,30,31]. TNFRSF9, expressed on activated CD4+/CD8+ T cells, NK cells, and antigen-presenting cells, modulates immune responses through CD137 signaling, promoting effector T cell expansion, cytokine secretion, and anti-apoptotic effects [32,33,34].

Our comprehensive analysis revealed a complex relationship between the LASSO-derived risk score and the tumor microenvironment (TME). Through the integration of the ESTIMATE algorithm and single-sample gene set enrichment analysis (ssGSEA), we observed significant alterations in immune and stromal components associated with risk stratification. High-risk GBM patients exhibited elevated stromal and immune scores, coupled with reduced tumor purity. Moreover, the immunosuppressive TME in GBM often reprograms infiltrating immune cells into pro-tumor phenotypes, including M2-polarized macrophages and Tregs [35], which suppress anti-tumor immunity and contribute to enhanced tumor invasiveness, immune escape, and therapeutic resistance [36].

The immune landscape of high-risk GBM is characterized by a paradoxical coexistence of cytotoxic and immunosuppressive components [4]. Treg signaling, M2 macrophage polarization, and immunosuppression mediated by inhibitory cytokines such as IL-10 and adenosine contribute to a “functional drowning” effect on CD8+ T cells, decoupling immune infiltration from clinical benefit and accelerating disease progression [37]. The Treg-M2 macrophage PD-L1 axis emerges as a critical immunosuppressive “vortex”, subverting anti-tumor immunity in high-risk GBM. Furthermore, MHC molecules play a crucial role in macrophage and CD8+ T cell interactions [38]. Macrophages express MHC class II molecules, which facilitate antigen presentation to CD4+ T cells, initiating immune responses [39]. Meanwhile, MHC class I molecules on nucleated cells present antigenic peptides for recognition by CD8+ T cells, thereby promoting cytotoxic activity against tumor cells [40,41]. Consistent with these findings, our study demonstrated a positive correlation between high-risk scores and increased MHC expression (Figure 5k–n).

In our study, we also found that there are two small-molecule compounds that are associated with different genes and have the potential for synergistic effects. I-BET-762 and Enzastaurin (LY317615) have potential associations with macrophages and major histocompatibility complex (MHC) class molecules. As a bromodomain and extra-terminal (BET) inhibitor, I-BET-762 can regulate the function and polarization of macrophages, inhibit the polarization of M2 macrophages, and reduce the secretion of immunosuppressive cytokines [42]. It may also indirectly affect the expression of MHC class molecules by regulating gene expression, thereby enhancing the antigen presentation function [43]. In the treatment of gliomas, I-BET-762 can synergize with MEK inhibitors to enhance the apoptotic response of rat and human glioma cells to HMBA both in vitro and in xenografts in vivo [44]. As a protein kinase C (PKC) inhibitor, Enzastaurin can affect the activation, phagocytosis, and cytokine secretion of macrophages [45]. Clinical studies have shown that when Enzastaurin is combined with bevacizumab for the treatment of recurrent malignant gliomas, patients have good tolerance, and the treatment response and progression-free survival are similar to those of bevacizumab monotherapy [46]. Although there is relatively little direct research on its relationship with MHC class molecules, given that the PKC signaling pathway is involved in various intracellular signal transduction processes, Enzastaurin may indirectly affect the functions of MHC class molecules in aspects such as antigen processing and presentation. However, the current research on the relationship between these two drugs and macrophages, as well as MHC class molecules, is still in the exploratory stage, and the specific mechanisms of action and the extent of influence may vary in different experimental models and disease contexts.

Although our study has successfully established a reliable prognostic risk scoring system related to the major histocompatibility complex (MHC), the existing limitations cannot be ignored. Due to the retrospective nature of this analysis, it is difficult to make causal inferences; therefore, prospective validation is urgently needed. At the same time, the relatively small sample size of normal brain tissue used for differential gene expression analysis may affect the statistical power. However, it is worth noting that the identified four-gene risk score has demonstrated high predictive accuracy and has been verified in multiple independent cohorts, which fully demonstrates its potential as a clinical prognostic tool. The nomogram generated based on this risk score provides convenience for clinicians to assess the survival probability of patients and make treatment decisions. In addition, the elucidation of the immune landscape associated with different risk stratifications provides important insights for the development of novel immunotherapeutic strategies targeting the immune escape mechanisms of patients with high-risk glioblastoma multiforme (GBM). Further research is required to clarify the direct role of these genes in the pathogenesis of glioblastoma and to deeply analyze the molecular mechanisms underlying their prognostic significance. It is worth mentioning that current research on the relationship between the small-molecule compounds I-BET-762 and Enzastaurin, macrophages, and MHC molecules is still in the exploratory stage. The specific mechanisms of action and the extent of influence vary among different experimental models and disease contexts, which will also become an important direction for in-depth research in the future.

## 4. Materials and Methods

### 4.1. Data Acquisition and Preprocessing

RNA sequencing (RNA-seq) gene expression data and corresponding clinical information for glioblastoma (GBM) patients were retrieved from the Cancer Genome Atlas (TCGA) database (https://portal.gdc.cancer.gov/ (accessed on 3 February 2025)) and the Chinese Glioma Genome Atlas (CGGA) database (https://www.cgga.org.cn/ (accessed on 3 February 2025)). The TCGA dataset served as the training cohort, while the CGGA dataset served as an independent validation cohort. Raw RNA-seq count data were preprocessed using standard bioinformatics pipelines. Specifically, genes with low expression (sum of counts ≤ 1 across all samples) were filtered out. Variance-stabilizing transformation (VST) normalization was then applied to account for differences in sequencing depth across samples. Principal component analysis (PCA) was performed to assess sample clustering and identify potential batch effects.

### 4.2. Differential Gene Expression (DEG) Analysis

Transcriptomic quantification profiles from glioblastoma multiforme cases were acquired through the TCGA multi-omics repository, constituting the discovery cohort for model development. This primary dataset underwent rigorous quality control measures, including batch effect correction and library size normalization prior to analytical implementation. Data preprocessing was performed to ensure quality and consistency. Differential expression analysis was conducted using DESeq2, comparing tumor (*n* = 168) and normal (*n* = 5) samples. Genes with |log2FC| > 1 and adjusted *p*-value < 0.05 were considered significantly differentially expressed. Using the ggplot2 R package and pheatmap R package, a volcano plot was generated to visualize the distribution of DEGs, and a heatmap was created to display the expression patterns of the top 60 DEGs (30 most upregulated and 30 most downregulated). Hierarchical clustering was performed to assess the separation between tumor and normal samples based on the identified DEGs.

### 4.3. Weighted Gene Co-Expression Network Analysis (WGCNA)

The WGCNA R package was employed to identify gene co-expression modules based on the expression data from DEGs and MHC ssGSEA scores. MHC-related genes were downloaded based on the Genecard database (https://www.genecards.org/ (accessed on 18 February 2025)). Prior to network construction, hierarchical clustering was performed to detect and remove potential outliers, ensuring high-quality input data. We determined the soft-thresholding power by examining the fit of the scale-free topology model and the mean connectivity in relation to different soft-threshold values. After careful analysis, we chose a power of 5 as the most suitable soft-thresholding value for building the co-expression network. Subsequently, we employed the WGCNA algorithm to detect gene modules that exhibited highly correlated expression patterns. As a result, we successfully identified a total of 12 gene co-expression modules. To better understand the relationships between these modules and traits, we created a heatmap for visualization.

### 4.4. LASSO Regression Analysis

To identify key prognostic genes associated with MHC-related traits in glioblastoma (GBM), we employed least absolute shrinkage and selection operator (LASSO) regression analysis. This technique was applied to the top 30 genes from the previously identified red module in the WGCNA analysis. The LASSO regression was performed using the glmnet package in R. To determine the optimal tuning parameter (λ), we utilized a 10-fold cross-validation approach, repeated multiple times to ensure robustness. The lambda.min value, which gives the minimum mean cross-validated error, was selected as the optimal λ. LASSO coefficient profiles were generated to visualize the change in coefficient values for all considered genes as a function of the log-transformed λ. Genes with non-zero coefficients at the optimal λ were identified as key prognostic genes. This process resulted in the selection of four MHC-related genes: TNFSF14, MXRA5, FCGR2B, and TNFRSF9.

### 4.5. Risk Score Calculation and Patient Stratification

Using the four-gene signature identified through LASSO regression, we calculated a risk score for each patient in both the TCGA training cohort and the CGGA validation cohort. Patients were stratified into high-risk and low-risk groups based on the median risk score within each cohort. Risk Score Calculation: Based on the LASSO regression results, we calculated a risk score for each patient using the following formula: Risk Score = Σ (Coefficient_i × Expression_i), where Coefficient_i is the LASSO coefficient for gene i, and Expression_i is the expression level of gene i. This risk score was used for subsequent survival predictions in GBM patients.

### 4.6. Survival Analysis

Kaplan–Meier survival analysis was performed to evaluate the prognostic value of the risk score model. Overall survival (OS) was compared between high-risk and low-risk groups using the log-rank test. Hazard ratios (HRs) with 95% confidence intervals (CIs) were calculated using Cox proportional hazards regression. Median survival times were determined for each risk group. The survival and survminer packages in R were used for these analyses.

### 4.7. Time-Dependent Receiver Operating Characteristic (ROC) Curve Analysis

To assess the predictive accuracy of the risk score model, we conducted time-dependent ROC curve analysis using the timeROC package in R. ROC curves were generated for 1-year, 2-year, and 3-year survival predictions. The area under the curve (AUC) was calculated for each time point to quantify the model’s discriminatory power. The x-axis of the ROC curve represents the false-positive rate, while the y-axis represents the true-positive rate.

### 4.8. Risk Score Distribution and Gene Expression Analysis

We visualized the distribution of risk scores and their relationship with survival status using a composite plot. The plot consists of three panels: (1) Risk Score Distribution: The top panel displays the distribution of risk scores for all patients, ordered from lowest to highest. (2) Survival Status: The middle panel shows the survival status of each patient, corresponding to their risk score. This allows for visual assessment of the relationship between risk score and mortality. (3) Gene Expression Heatmap: The bottom panel presents a heatmap of the expression levels of the four model genes for each patient. Red indicates high expression, while blue indicates low expression. Patients are ordered by increasing risk score from left to right. This composite plot was generated using the pheatmap package in R, with custom modifications to incorporate the risk score distribution and survival status information.

### 4.9. Validation in Independent Cohort

To validate the robustness and generalizability of our risk score model, we applied the same analytical procedures to an independent cohort from the Chinese Glioma Genome Atlas (CGGA). This included recalculating risk scores, performing Kaplan–Meier survival analysis, conducting time-dependent ROC curve analysis, and generating the risk score distribution plot.

### 4.10. Gene Ontology and KEGG Pathway Enrichment Analyses

To elucidate the biological context of the identified DEGs, we conducted Gene Ontology (GO) and Kyoto Encyclopedia of Genes and Genomes (KEGG) pathway enrichment analyses. The clusterProfiler package in R was utilized for these analyses. For GO analysis, we focused on biological process terms, while KEGG analysis encompassed all available pathways. Enrichment was considered significant at an adjusted *p*-value < 0.05. Results were visualized using bar plots, with the length of bars representing gene counts and color intensity indicating statistical significance.

### 4.11. Gene Set Enrichment Analysis (GSEA)

GSEA was performed to identify biological pathways associated with the LASSO risk score. We used the fgsea package in R, with genes ranked based on their correlation with the risk score. Enrichment was calculated against the Molecular Signatures Database (MSigDB) hallmark gene sets. Significance was determined using an adjusted *p*-value < 0.05 and false discovery rate (FDR) < 0.25. The top 10 significantly enriched pathways were visualized using enrichment plots, highlighting the leading-edge genes.

### 4.12. Screening of Small Molecule Drugs and Sensitivity Calculation

Information on the expression of small-molecule drugs and related indices can be obtained from the RNAactDrug database. The structural diagrams of small molecules are were obtained from the Chemical Biology Database (CchEMBL, https://www.ebi.ac.uk/ (accessed on 15 January 2025)) of the European Bioinformatics Institute (EBI) (Cambridge, UK) and the Connectivity Map Program Database (HMC LINCS, https://lincs.hms.harvard.edu/ (accessed on 17 January 2025)) of Harvard Medi-cal School (Boston, MA, USA). The drug sensitivity data is were obtained and calculated from the Cancer Rx Gene Database (Cancerrxgene, https://www.cancerrxgene.org/ (ac-cessed on 20 January 2025)).

### 4.13. Gene–Gene Interaction Network Analysis

To explore functional interactions among the four key genes (TNFSF14, MXRA5, FCGR2B, and TNFRSF9), we constructed a gene–gene interaction network using GeneMANIA. This web-based tool integrates multiple databases to predict gene functions and visualize gene interactions based on co-expression, physical interactions, and shared protein domains.

### 4.14. Immune Cell Infiltration Analysis

To comprehensively assess the relationship between LASSO risk scores and immune cell infiltration, we employed two complementary computational approaches: ESTIMATE and single-sample gene set enrichment analysis (ssGSEA).

#### 4.14.1. ESTIMATE Analysis

We made use of the estimation of stromal and immune cells in malignant tumor tissues using the expression data (ESTIMATE) algorithm to deduce the proportion of stromal and immune cells within tumor samples. This algorithm was implemented on the normalized gene expression data. As a result, it produced stromal scores, immune scores, and combined ESTIMATE scores for every sample. The samples were divided into high-risk and low-risk groups according to the median LASSO risk score. Then, we compared the scores between these two groups.

#### 4.14.2. ssGSEA Analysis

Single-sample gene set enrichment analysis (ssGSEA) was performed to evaluate the enrichment of specific immune cell populations within individual samples. We used established gene signatures for various immune cell types, including CD8+ T cells, NK cells, and T regulatory cells (Tregs). The ssGSEA algorithm was implemented using the GSVA package in R, with enrichment scores calculated for each immune cell type across all samples.

### 4.15. Gene Expression and Survival Analysis

#### Differential Expression Analysis

We analyzed the expression profiles of TNFSF14, MXRA5, FCGR2B, and TNFRSF9 in glioblastoma multiforme (GBM) tissues compared to normal brain samples. Gene expression data were obtained from the Cancer Genome Atlas (TCGA). Expression levels were quantified as log_2_(TPM+1) values, where TPM represents Transcripts Per Million. Differential expression between GBM and normal samples was assessed using unpaired *t*-tests, with statistical significance set at *p* < 0.05.

### 4.16. Immunohistochemical Analysis

To validate our transcriptomic findings at the protein level, we examined immunohistochemical (IHC) staining data for TNFSF14 and MXRA5 in GBM and normal brain tissue specimens. IHC images were obtained from the Human Protein Atlas (https://www.proteinatlas.org (accessed on 5 March 2025)), an open-access database of protein expression profiles in human tissues.

### 4.17. Statistical Analysis

For all three approaches, comparisons between high- and low-risk groups were conducted using the Mann–Whitney U test. *p*-values were adjusted for multiple comparisons using the Benjamini–Hochberg method to control the false discovery rate. Statistical significance was set at adjusted *p* < 0.05. All statistical analyses were performed in R (version 4.0.3). For all analyses, a *p*-value < 0.05 was considered statistically significant unless otherwise specified.

## Data Availability

The datasets generated, coded, and analyzed during the current study are available upon request.

## Supplementary Materials

The following supporting information can be downloaded at: https://www.mdpi.com/article/10.3390/ijms26104609/s1.

## Abbreviations

The following abbreviations are used in this manuscript:

TCGA	The Cancer Genome Atlas
CGGA	Chinese Glioma Genome Atlas
WGCNA	Weighted gene co-expression network analysis
LASSO	The least absolute shrinkage and selection operator
MHC	Major histocompatibility complex
