# Supplementary material for: An MHC-Related Gene’s Signature Predicts Prognosis and Immune Microenvironment Infiltration in Glioblastoma
Source: Int J Mol Sci. 2025 May 12;26(10):4609. doi: 10.3390/ijms26104609 (PMC12111048; doi:10.3390/ijms26104609)
Supplement: Supplementary file 1 [file ijms-26-04609-s001.zip › ijms-3585719-supplementary.pdf]

Article

An MHC-Related Gene's Signature Predicts Prognosis and Immune Microenvironment Infiltration in Glioblastoma

Caiyuan Yu [1,2,3,†], Mingjuan Xun [2,†], Fei Yu [1], Hengyu Li [1], Ying Liu [1], Wei Zhang [1,*] and Jun Yan [2,*]

1 School of Pharmacy, Faculty of Medicine & State Key Laboratory of Quality Research in Chinese Medicines, Macau University of Science and Technology, Macau SAR 999078, China; yucy@ouchn.edu.cn (C.Y.); 3220004386@student.must.edu.mo (F.Y.); 3220005009@student.must.edu.mo (H.L.); yingliu@xhsysu.cn (Y.L.)

2 Laboratory of Brain Disorders, Beijing Institute of Brain Disorders, Ministry of Science and Technology, Collaborative Innovation Center for Brain Disorders, Capital Medical University, Beijing 100069, China; mingjuan_immunity@163.com

3 College of Agroforestry and Medicine, The Open University of China, Beijing 100039, China

* Correspondence: wzhang@must.edu.mo (W.Z.); yanjun@ccmu.edu.cn (J.Y.)

† These authors contributed equally to this work.

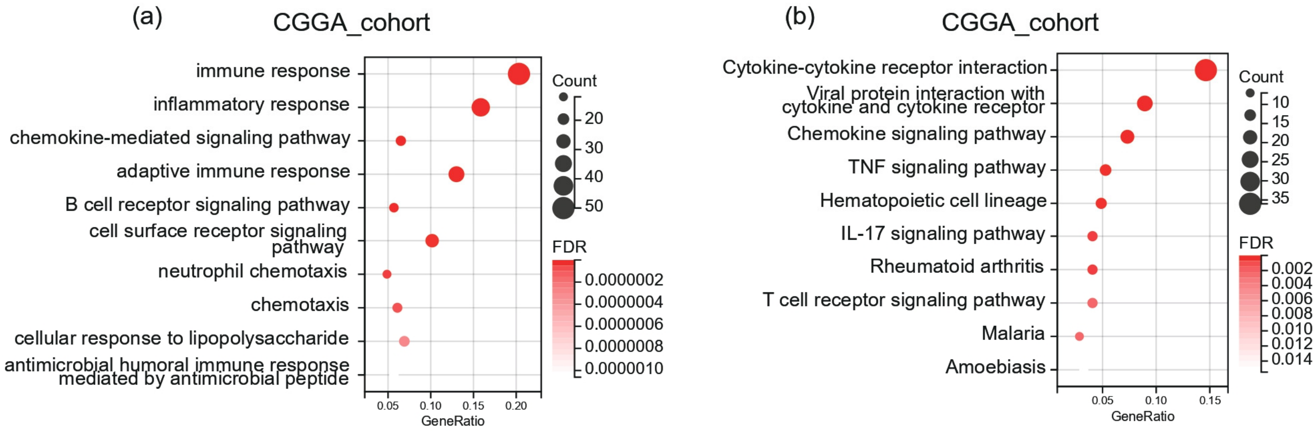

Figure S1. The main functional pathways significantly correlated with riskscore in CGGA cohort. (a, b) Gene Ontology (GO) enrichment analysis and Kyoto Encyclopedia of Genes and Genomes (KEGG) pathway analysis in CGGA cohort.
